# Immune response in influenza virus infection and modulation of immune injury by viral neuraminidase

**DOI:** 10.1186/s12985-023-02164-2

**Published:** 2023-08-28

**Authors:** Hongyu Jiang, Zongde Zhang

**Affiliations:** 1 The People’s Hospital of Dayi Country, Chengdu, Sichuan China; 2 Inflammation and Allergic Diseases Research Unit, The Affiliated Hospital of Southwest Medical University, Luzhou, Sichuan China; 3 School of Basic Medical Sciences, Southwest Medical University, Luzhou, Sichuan China

**Keywords:** Influenza virus, Neuraminidase, Cytokine storm, CD83, Immune response

## Abstract

Influenza A viruses cause severe respiratory illnesses in humans and animals. Overreaction of the innate immune response to influenza virus infection results in hypercytokinemia, which is responsible for mortality and morbidity. The influenza A virus surface glycoprotein neuraminidase (NA) plays a vital role in viral attachment, entry, and virion release from infected cells. NA acts as a sialidase, which cleaves sialic acids from cell surface proteins and carbohydrate side chains on nascent virions. Here, we review progress in understanding the role of NA in modulating host immune response to influenza virus infection. We also discuss recent exciting findings targeting NA protein to interrupt influenza-induced immune injury.

## Introduction

Influenza is highly contagious, and acute viral respiratory infections may occur as pandemics, epidemics, and outbreaks [1–3]. The susceptibility to infection involvement in all age stages, prominent individuals with chronic comorbid diseases, immunosuppression, pregnant and postpartum women, and frail older adults; most commonly occurs in spring and winter, and the virus causes significant mortality and morbidity worldwide [2, 4, 5]. Influenza viruses belong to the Orthomyxoviridae RNA virus and classify into four distinct types based on their antigenic differences: influenza A, influenza B, influenza C, and influenza D. Influenza A viruses infect a broad range of host species. However, the main hosts are humans for influenza B and C, and influenza D viruses have not infected humans. Influenza A is the type most responsible for causing pandemics because of its high susceptibility to antigenic variation [6–8], so we will mainly discuss understanding influenza A viruses (IAV) infection.

IAV virions are constructed from a host cell-derived membrane and various viral proteins [9, 10]. Eight single-stranded viral RNA segments for 10 structural and at least 9 nonstructural/regulatory proteins. PB1, PB2, PA, NP, M1, NS1, and NEP are inside the lipid envelope. At the same time, M2, Hemagglutinin (HA), and Neuraminidase (NA) are embedded in the envelope and available for antibody binding [10–15]. IAV infects the upper respiratory tract at first, enters epithelial cells through endocytosis, and infects the lower respiratory tract with the disease developed [16, 17]. The HA protein binds to sialic acid residues expressed in the airway or alveolar epithelium, triggering the endocytosis of viral particles [18]. The virus completes the shedding, assembly, and release on the membrane. In the procession of viral shedding, NA cuts the connection between the HA of newly formed viral particles and sialic acid receptors on the cell surface, releasing progeny viruses, which promotes viral replication, transcription, and translation, infecting neighboring cells or leaving the individual through respiratory droplets [19, 20]. For the past few years, the widespread use of NA inhibitors has raised the concern about drug resistance. Here, we review the role of NA in modulating the host immune response to influenza virus infection.

## Neuraminidase: structure, mutation, and function

### NA structure

Neuraminidase, located on the virus's surface and belongs to glycoproteins, next to HA and matrix protein2(M2), is also called sialidase [21]. NA is a tetramer of four identical polypeptides, presenting the mushroom-like structure consisting of four domains: an N-terminal cytoplasmic sequence, followed by a membrane-anchoring hydrophobic transmembrane domain (TMD) and a thin stalk of variable length, ending in a globular head domain, the binding site for sialic acid is located in the head domain [21–24]. Each protomer comprises approximately 470 amino acid residues [23]. NA tetramer dimensions about 10 nm × 15 nm, cleaves sialic acid for virion release 20–50 tetramers per spherical virion. Space for about 13 bound Fabs per tetramer, containing 1880 aa/tetramer, weight 220 kDa/tetramer for prototypical lab-adapted strains of the influenza A virus [25].

The N-terminal cytoplasmic domain has six amino acid residues (MNPNQK); this sequence is nearly 100% conserved across all influenza A subtypes. The arrangement has been highly preserved in influenza A and B viruses [26, 27]. The cytoplasmic domain is also essential for attaching NA with lipid rafts [19].

The TMD can transport newly expressed NA to the apical plasma membrane with the N-terminal cytoplasmic tail [28]. The TMD that follows the short cytoplasmic sequence is variable in sequence among subtypes. Still, all subtypes form a transmembrane helix encompassing amino acids 7–29 when analyzed by the highly reliable program TMHMM [29–31].

The stalk domain connects the TMD with the catalytic head domain. The NA stalk varies in length within and across NA subtypes and contains multiple predicted N-linked glycosylation sites. And, with few exceptions, it has at least one cysteine residue that can form an intermolecular disulfide bond with a neighboring NA molecule. Glycosylation of the stalk region may contribute to NA stability; the disulfide bond needs to format tetramer [31]. A discovery indicated that specificity and affinity to sialic acids by the HA is highly dependent on sugar conformation and extension, and the stalk length of NA can impact combining HA with sialic acids [31–35].

**Fig. 1:**
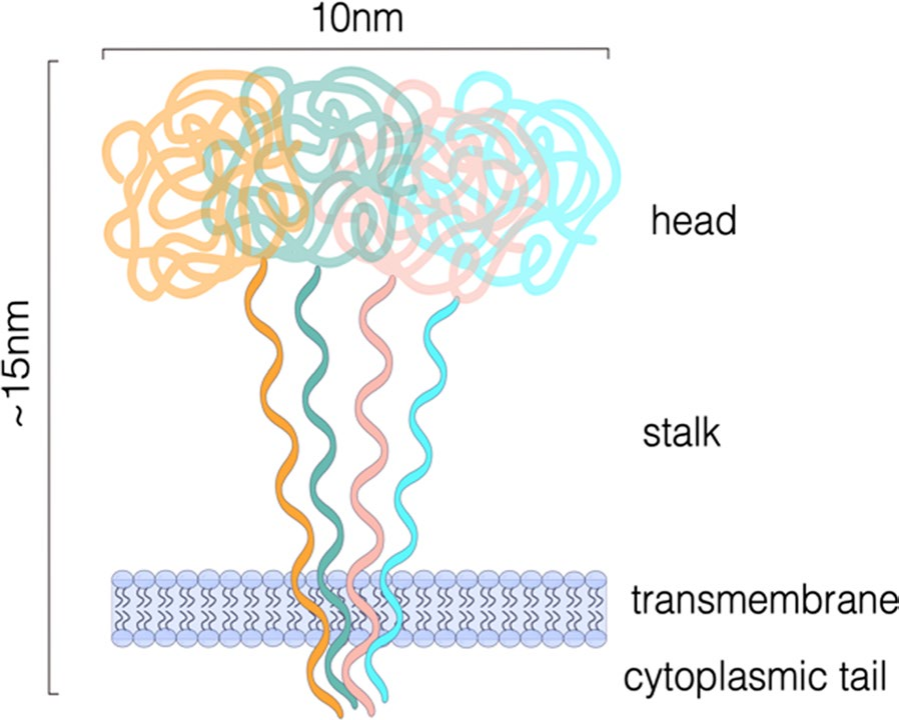
Neuraminidase structure. Neuraminidase is comprised of N-terminal cytoplasmic tail, transmembrane, NA stalk and NA head. A tetramer dimensions about 10 nm × 15 nm from snapshot

The NA head domain is characterized by a six-bladed propeller folded around the catalytic site and is typical for all known sialidases [36]. Each blade comprises four antiparallel beta sheets stabilized by disulfide bonds and is connected by variable-length loops [31]. In of to classify with glycoproteins, NA possess nine different classes, N1–N9, crystal structures of the head domain of at least one representative NA from N1 to N9 and from influenza B NA have been analyzed, and crystal structures of NA encompass the catalytically active heads [36–39] (Fig. 1).

### NA mutation

Influenza viruses evolve quickly through frequent antigenic variation. Antigenic drift and shift are terms used to describe how the virus mutates and results in new strains. Drastic changes in the antigenicity of the HA of circulating influenza A viruses is called antigenic shift; the animal strains of the influenza virus can be acquired by human influenza strains through reassortment [40]. We and others established a mice model for H9N2 infection, which revealed multiple amino acid substitutions in NA related to enhanced virulence in mice [41, 42]. There is a significant change in the virus's genome in antigenic shift resulting in new HA and NA protein expression [1, 43], which can cause a medium or small epidemic [44]. Influenza viruses can evade the antibody-mediated immunity induced during infections or vaccinations by gradually accumulating mutations in HA and NA, known as antigenic drift [40]. The antigenic drift of HA has been extensively studied [45, 46]. By using neuraminidase inhibition assays, the antigenic drift of NA mostly matches between vaccines and circulating viruses [40, 47, 48]. The reassortment and evolution of NA and linked HA may result in an antigenic drift of both significant surface glycoproteins, reducing vaccine efficacy and subsequently impacting animal health [49]. K199 and E258 mutations significantly affected Mab binding, NA inhibition, and neutralization. The activity and the modifications help detect antigenically drifted NAs [50]. A less obvious location for functional variation is the fibrous stalk that attacks the globular domain to the membrane, governs the length and the height of the globular domain, and hence its access to substrates and its interactions with HA [51, 52]. However, influenza viruses circulate in different species, such as birds to humans. Subtype antigenic variation is limited, so vaccines can also be selected limited.

### NA function

Robust protective human immunity against influenza is primarily provided by antibodies targeting the virus's variable epitopes, those found on portions of its surface glycoproteins. When influenza viruses infect the body, HA mediates binding to sialic acids on host cell glycoproteins or lipids. Then fusion of the host cell and virus membrane through a low pH-induced irreversible conformational changes, primarily as HA2 anchors HA in the envelope and is directly involved in membrane interaction [53]. HA is essential in the entry process. NA is which sialidase catalyzes the removal of the terminal sialic acids. As IAV is essentially reversibly bound in the NA activity, motility enables virion penetration of the mucus layer by cleaving sialic acids as well as attachment to and uptake into the epithelial cells of the respiratory tract, so that infect underlying epithelial cells [54, 55]. Studies show that the enhancement of fusion and infectivity by NA was related to the sialylation of virion-expressed HA, so NA activity plays a critical role in virion infectivity and HA-mediated membrane fusion [56]. On the other hand, the influenza replication cycle needs to release the least newly formed virions from the infected cell and prevent virion aggregation by removing sialic acid from the viral and host cell membrane [57]. Currently, many NA inhibitors are discovered by structure–activity relationship, and these inhibitors fight against the surge in resistance resulting from naturally occurring mutations.

## Cytokine storm

The IAV infects epithelial cells, endothelial cells, and alveolar macrophages to produce the first wave of cytokines, especially type I interferons (IFNs), which upregulate the expression of numerous IFN-stimulated genes (ISGs).

Following the type I IFNs released, higher expression of ISGs initiates downstream antiviral responses and subsequent inflammatory cytokine production by innate immune cells. Then the adaptive immune cells (different subsets of T cells and group 2 innate lymphoid cells) are activated and regulated to secrete the second wave of cytokines that promote viral clearance, tissue homeostasis, and lung repair [58]. The innate immune response is regulated by chemokines and cytokines, chemical messengers produced by virus-infected epithelial cells and leukocytes [59], and natural interferon-producing cells, such as plasmacytoid dendritic cells [60]. A study found that the only producers of antiviral cytokines were infecting epithelial cells; plasmacytoid dendritic cells were potent producers of IFNs in the body by using an animal model [61]. After influenza viruses infect the host, the IAV first induces the innate immune system, which can rapidly recruit innate immune cells and cytokines to the site of infection [62]. Cytokines are essential for intercellular communication and viral clearance in the immune system, but excessive cytokines can cause severe immune pathology. Excessive production of pro-inflammatory cytokines leads to aggressive proinflammatory responses. The insufficient control of anti-inflammatory responses is called a cytokine storm or hypercytokinemia, which causes significant immunopathology and serious disease consequences, such as acute respiratory distress syndrome (ARDS) [17, 63–68].

From a pathology perspective, the characteristic alveolar changes of influenza-virus pneumonia caused by cytokine storms include capillary thrombosis, focal necrosis, congestion of the alveolar wall, hyaline membrane formation, pulmonary edema, peribronchial hemorrhage, peribronchial pneumonia [69]. The changes characteristic of severe influenza viral pneumonia include capillary and small vessel thromboses, interstitial edema and inflammatory infiltrate, the formation of hyaline membranes in alveoli and alveolar ducts, varying degrees of acute interalveolar edema and hemorrhage, and diffuse alveolar damage in addition to necrotizing bronchitis and bronchiolitis. In later stages of diffuse alveolar damage, fibrosis, epithelial regeneration, and squamous metaplasia [69, 70]. Severe cytokine storms can cause multiple organ dysfunction syndromes, systemic inflammation, and even death [17, 71–73]. Cytokine storms can lead to host immune response disorders, primarily the innate immune system, and can cause lung damage after the influenza virus infects the body. Many studies have shown that many factors are related to NA modulating host immune response to influenza virus infection.

Biological factors may affect the host's susceptibility to the influenza virus and its anti-immune response. Activated macrophages were the cellular source of cytokines and chemokines in young and old mice. Macrophages, dendritic cells, and NK cells are activated in younger mice. Dendritic cells did not have this same effect in older mice. The cellular source of many cytokines and chemokines shifted as aging [74]. The different ways and times of vaccine injection also affect immune protection [75]. The risk of influenza-related death increases exponentially after age 65, with over 90% of the annual influenza virus-related mortality from this age stage group in the United States [76]. The severe consequences of the influenza virus infection in children are related to the cytokine storm [77–79]. The airway epithelial TLR3 drives IFN-β production in response to IAV infection, as determined by genetic mapping of TLR3-associated mutations in children who acquire severe IAV-induced ARDS [80]. Therefore, cytokine storm is likely related to age, affecting immune response.

Obesity is an independent risk factor for increased disease severity and death during IAV infection. Obesity primes the innate immune system to respond to IAV with a heightened proinflammatory response and a blunted antiviral response, leading to increased tissue damage and decreased virus elimination [81]. Immune response to infection is impaired in obese individuals [82]. B cells exacerbate inflammation and insulin sensitivity by producing auto-antibodies in fat mice [83]. Increased inflammation, particularly elevated IL-6 levels, activation of Renin–angiotensin–aldosterone system (RAAS), rise in Angiotensin II(Ang II) levels, higher leptin, and increased ectopic fat favor influenza virus progression and severity [84]. Therefore, cytokine storm is related to obesity.

Influenza viruses rely on numerous host factors to support their replication [85–88]. Sialic acid is a determinant of the host range. Viral neuraminidase displays species-specific adaptation. Different expression patterns of detection and antiviral effector molecules in other species will drive the adaption of influenza viruses when they infect a new host. This adaption can involve changes that alter the binding partners and the relative expression or cellular location of the viral antagonist of the cellular innate immune response [89]. Influenza induces DNA damage, and DNA damage responses are activated; the host response causes DNA damage in lung epithelium after influenza infection; DNA repair modulates the severity of influenza-induced cytotoxicity, thereby affecting tissue damage and regeneration [90]. Targeting host factors involved in virus replication and controlling virus-induced host immune responses [91]. The extent of cellular coinfection by influenza viruses may be a critical determinant of both viral production kinetics and cellular infection outcomes in a host cell type-dependent manner [92]. Therefore, host factors can affect the host's immune response after an influenza virus infection.

Endothelial cells are central regulators of cytokine storms during influenza virus infection [93]. Vulnerability to secondary bacterial infection peaks at approximately one-week post-influenza infection; influenza virus infection facilitates secondary bacterial infection through phagocyte function (macrophage and neutrophil) or phagocyte-independent mechanisms, regulation of antimicrobial peptide, expression of IFN, immune cells (Th17 cells, NK cells, Treg cells, iLCs), and genetic susceptibility [94]. Studies indicate that wound healing was delayed when mice with healing wounds were infected with IAV in the lung; an inflammatory cytokine milieu characterizes the earliest phase of cutaneous wound repair. The viral lung infection suppresses the innate immune response in a healing wound, including cellular infiltrate, chemokines, growth factors, and cytokines; the cytokine and chemokine expression indicates a lung infection can induce changes in the dermal wound environment of cutaneous and subcutaneous wounds [95–98]. Phospholipids present in the pulmonary surfactant complex, palmitoyl-oleoyl-phosphatidylglycerol (POPG) and phosphatidylinositol (PI) could disrupt the virus particle binding to host cell plasma membrane receptors [99]. The antagonism of activation of TLRs and virus binding to the alveolar epithelium by resident constituents of the pulmonary surfactant system suggests that POPG and PI function in homeostasis to prevent inflammatory processes that result in reductions in gas exchange within the alveolar compartment. Antagonism of TLR activation inhibits proinflammatory signaling pathway initiation steps; lipids block TLR recognition of activated ligands directly or through TLR4 co-receptors cluster of differentiation 14(CD14) and Myeloid differentiation protein 2 (MD2) [99]. Therefore, boosting lipid synthesis or increasing the expression of CD14 and MD2 can inhibit cytokine production in the lung. A study indicates that upregulated expression of cellular adhesins by TGF-β, especially fibronectin-binding protein activated in influenza viral infection, increases host susceptibility to secondary bacterial pneumonia or coinfection [100]. Therefore, reducing fibronectin-binding protein expression and TGF-β production, which cannot cause secondary bacterial pneumonia and coinfection.

**Fig. 2:**
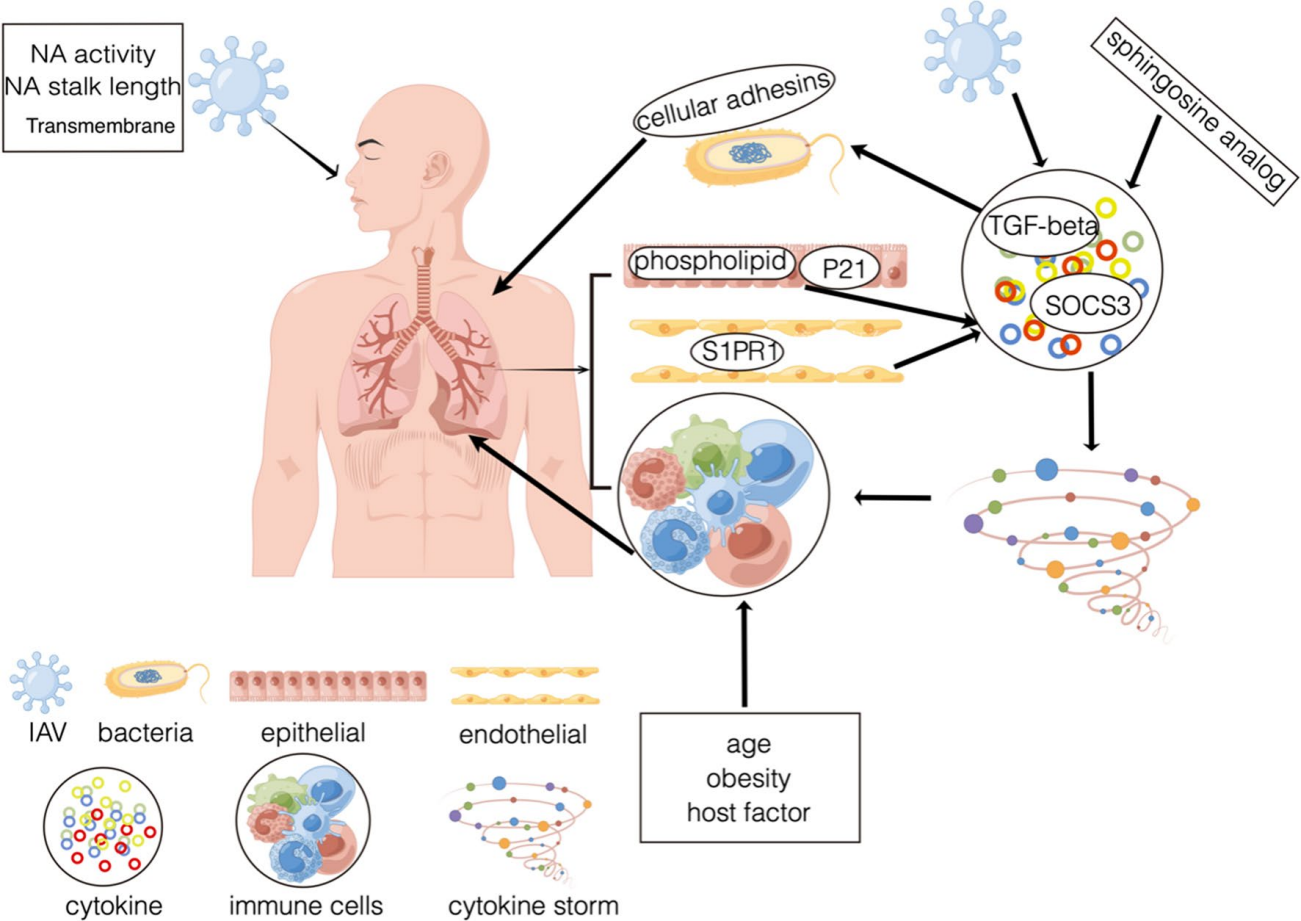
The relationship of virus, immunity response, cytokine and lung. (1) Factor: from virus (NA activity, NA stalk length and transmembrane), from host (age, obesity and host factor), and these factors can affect immune response; (2) Phospholipid and p21 locate in the pulmonary epithelium, S1PR1 locate in the pulmonary endothelium, SOCS3 as inhibitory cytokine, pulmonary endothelium is central orchestrator of cytokine amplification. When virus invade body, p21 expression reduced, SOCS3 and S1PR1 signaling improved, immune cells are activated, frequent the number of cytokines is reduced, lung injury was decreased; (3) When body is infected with influenza virus, phospholipid increased, then the number of cytokines is increased, immune cells are inhibited, lead to cytokine storm and finally cause lung injury; (4) When virus invaded, TGF-β is activated through virus NA, then upregulated expression of cellular adhesins, immune cells are inhibited, and lead to cytokine storm, cause bacterial pneumonia

Influenza virus infections are associated with a cytokine storm and an exaggerated innate immune response [101]. A study indicates that p21 restricts IAV by perturbing the viral polymerase complex and activating the host's natural immune response. p21 directly interacts with HP-1 to inhibit K-48 ubiquitination-mediated degradation after IAV infection. p21 promotes IRF3 activation via the recruitment of HO-1 through the inhibition of K48-linked ubiquitination degradation, resulting in increased expression of type I IFNs. P21 acts as a positive regulator of type I IFN during IAV infection, a new role in innate immunity [102]. Therefore, enhancing p21 expression restricts the influenza A virus. A study found that disruption of SOCS3 expression provided significant protection against IAV infection in IAV early disease, attenuated acute lung injury, and silenced SOCS3 enhanced STAT3 activity and regulated NF-κB and IL-6 so that IAV circumvent IL-6/STAT3-mediated immune response through upregulating SOCS3 [103]. Therefore, disrupting influenza virus infection by restricting SOCS3 expression. Sphingosine 1-phosphate receptor 1 (S1PR1) is expressed by lymphocytes and endothelial cells and is known to control lymphocyte egress from lymph nodes [104]. Sphingosine 1-phosphate (S1P) agonist therapy suppresses innate immune cell recruitment and cytokine-chemokine production. S1P therapy could suppress detrimental innate immune responses without hindering virus control [93, 105]. Sphingosine analog AAL-R inhibits cellular and cytokine/chemokine responses and activates natural inflammatory infiltrate [105]. S1PR1 agonist is through inhibition downstream of myeloid differentiation primary response gene 88 and IFN-β promoter stimulator-1 signaling for blunting cytokine storm [106]. Therefore, activating S1PR1 signaling or using sphingosine analog to blunt cytokine storm for protecting the infected host from the consequences of influenza infection (Fig. 2).

## Targeting neuraminidase and NA-CD83 mediate the immune response

Recently, more and more research has shown that targeting NA helps suppress influenza virus infection. NA inhibitors are licensed as influenza therapeutics inhibiting NA activity [107–110]. KW is derived from the brown algae *Kjellmaniella crassifolia*, which blocks IAV invasion and release by targeting viral NA and cellular EGFR pathways [111]. Eliciting neutralizing antibodies that recognize variable epitopes on the HA head is the dominant way influenza vaccines protect individuals from influenza and prevent the spread of influenza through populations. Adjuvanted vaccines induce more robust CD4+ T cells and prolific germinal center reactions and activate naïve B cells with new specificities [112, 113]. By modulating NA stalk length in recombinant IAVs, anti-stem Abs inhibit virus release from infected cells by blocking NA, accounting for their in vitro neutralization activity. NA inhibitors enhance anti-stem-based Fc-dependent immune cell activation, raising the possibility of therapeutic synergy between NA inhibitors and anti-stem mAb treatment in humans, extending the NA stalk to enhance immunogenicity [114, 115]. A study found isolating three clonally related mAbs that bind to the influenza virus NA by inserting a long CDR H3 into the enzymatic active site, occupying the sialic acid substrate site and inhibiting all influenza A virus NA subtypes and influenza B virus NA [116]. NA is an important and protective antigen. NA is a promising target for future influenza vaccines, based on immunity optimally to enhance the breadth of influenza virus vaccines and increase vaccine efficacy [117–119]. A study found that it creates an NA inhibitor zanamivir-targeted cytotoxic drug and a viral NA-targeted CAR T cell, which can kill viral NA-expressing cells without damaging healthy cells [120]. The recombinant neuraminidase surface glycoprotein can enhance and broaden protection against the influenza virus [121]. Anti-NA antibodies are less dependent on the HA due to receptor binding for helping aid in the control of viruses [122–124]. Improving vaccine design for identifying NA antigenic drift and novel epitopes of anti-NA antibodies [125]. Moreover, the Chinese medicinal herb and compound could inhibit the influenza virus by targeting NA [109, 126, 127].

**Fig. 3:**
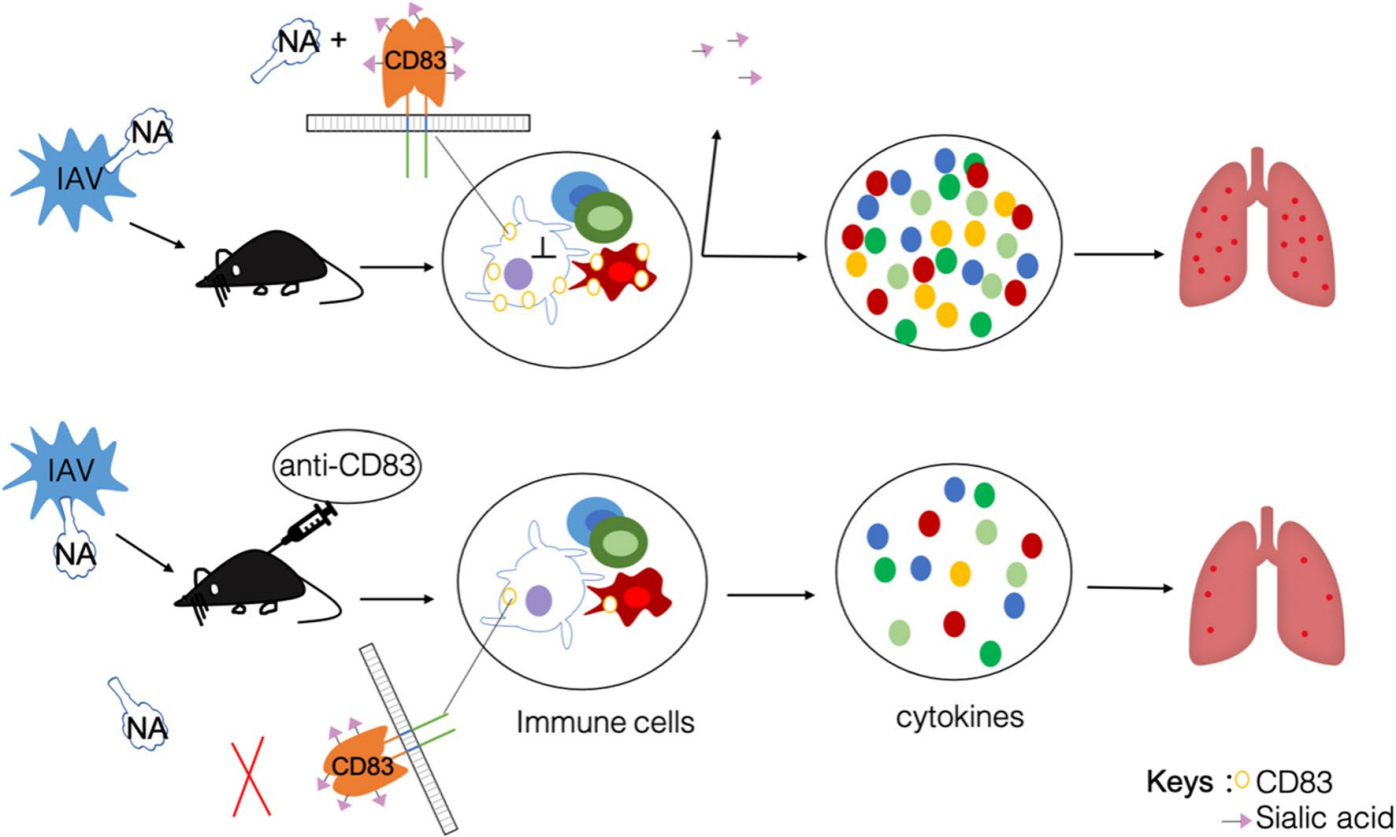
Neuraminidase-CD83 axis. When mice were infected with influenza virus, mainly component is neuraminidase, CD83 is a sialylated protein and sialylated CD83 delivers inhibiting signaling to DCs, CD83 expression level was upregulated on dendritic cells and macrophages in the lung. NA removed sialic acid and released superfluous cytokines, frequent causing lung injury; when using anti-CD83Ab for restrained NA across, reduced cytokines production, and reduction of lung injury

CD83 is a type I transmembrane glycoprotein expressed in mature dendritic cells. It is an activation marker for DCs and has been suggested to be a sialic acid-binding Ig-like lectin adhesion receptor. It is involved in two forms: membrane-bond CD83(mCD83) and soluble CD83(sCD83), mCD83 regulates maturation, activation, and homeostasis, sCD83 have an immune suppressive function [128–131]. CD83 regulates lymphocyte maturation, activation, homeostasis, and antibody response to immunization and infection [132]. CD83 in lymphocyte homeostasis and antibody production during IAV infection [133]. DCs infected with the influenza virus upregulate proinflammatory cytokines, including CD83, and simultaneously downregulate anti-inflammatory cytokines [134]. We previously used the influenza H9N2 virus-infected mice model and found that NA treatment directly increased CD83 on the cell membrane surface of DCs and enhanced NF-κB signaling. We prove that CD83 is a sialylated protein embedded and masked in the cell membrane. Sialylation of CD83 delivers inhibiting signaling to DCs. NA deactivates the regulatory CD83 pathway by removing sialic acid and releasing excessive cytokines, causing lung injury. Antibody blocking CD83 prevents NA access, or soluble CD83 decoy NA, can mitigate cytokine storms, reduce lung injury induced by the influenza virus, and alleviate influenza symptoms [135] (Fig. 3). The NA-CD83 axis may serve as a new potential target for treating the influenza virus.

## Conclusion

In recent years, interest has been aroused in understanding the immuno-modulating mechanisms of influenza virus neuraminidase. NA was a sialidase enzyme that cleaves sialic acids from cell surface proteins and carbohydrate chains on nascent virions. Cytokines are essential for intercellular communication and viral clearance in the immune system, but excessive cytokines can cause severe immune pathology. There are many medications available to prevent and protect against influenza viruses. Cytokine storm is responsible for mortality and morbidity. From virions structure, biology factors (age, obesity), host factor (DNA damage), lung (endothelial cells, lipid, pulmonary surfactant system), and cytokines/chemokines, these factors are involved in affecting immune response. The targeted NA through NA stalk length, NA activity, vaccines, anti-Ab antibodies, and so on. CD83 controls T cell and B cell maturation and regulates immune activity. NA upregulates CD83 expression in DCs and NA deactivates the regulatory CD83 pathway by removing sialic acid and releasing excessive cytokines, causing lung injury, so the path may inform novel and potential clinical strategies to target influenza virus pathogenesis strategically.

## Data Availability

Not applicable.
